# Is One Trial Sufficient to Obtain Excellent Pressure Pain Threshold Reliability in the Low Back of Asymptomatic Individuals? A Test-Retest Study

**DOI:** 10.1371/journal.pone.0160866

**Published:** 2016-08-11

**Authors:** Romain Balaguier, Pascal Madeleine, Nicolas Vuillerme

**Affiliations:** 1 Univ. Grenoble-Alpes, EA AGEIS, Grenoble, France; 2 Physical Activity and Human Performance group—SMI, Dept. of Health Science and Technology, Aalborg University, Aalborg, Denmark; 3 Institut Universitaire de France, Paris, France; University of Illinois-Chicago, UNITED STATES

## Abstract

The assessment of pressure pain threshold (PPT) provides a quantitative value related to the mechanical sensitivity to pain of deep structures. Although excellent reliability of PPT has been reported in numerous anatomical locations, its absolute and relative reliability in the lower back region remains to be determined. Because of the high prevalence of low back pain in the general population and because low back pain is one of the leading causes of disability in industrialized countries, assessing pressure pain thresholds over the low back is particularly of interest. The purpose of this study study was (1) to evaluate the intra- and inter- absolute and relative reliability of PPT within 14 locations covering the low back region of asymptomatic individuals and (2) to determine the number of trial required to ensure reliable PPT measurements. Fifteen asymptomatic subjects were included in this study. PPTs were assessed among 14 anatomical locations in the low back region over two sessions separated by one hour interval. For the two sessions, three PPT assessments were performed on each location. Reliability was assessed computing intraclass correlation coefficients (ICC), standard error of measurement (SEM) and minimum detectable change (MDC) for all possible combinations between trials and sessions. Bland-Altman plots were also generated to assess potential bias in the dataset. Relative reliability for both intra- and inter- session was almost perfect with ICC ranged from 0.85 to 0.99. With respect to the intra-session, no statistical difference was reported for ICCs and SEM regardless of the conducted comparisons between trials. Conversely, for inter-session, ICCs and SEM values were significantly larger when two consecutive PPT measurements were used for data analysis. No significant difference was observed for the comparison between two consecutive measurements and three measurements. Excellent relative and absolute reliabilities were reported for both intra- and inter-session. Reliable measurements can be equally achieved when using the mean of two or three consecutive PPT measurements, as usually proposed in the literature, or with only the first one. Although reliability was almost perfect regardless of the conducted comparison between PPT assessments, our results suggest using two consecutive measurements to obtain higher short term absolute reliability.

## Introduction

Pain is defined as an unpleasant sensory and emotional experience associated with actual or potential tissue damage, or describe in terms of such damage [1]. According to the American Pain Society [2], pain is the fifth vital sign of medical examination. Pressure algometry (PA) performed with a handheld algometer is a method increasingly used since the 80s to assess mechanical pain sensitivity in different anatomical regions. When it is applied perpendicularly to the skin, the algometer creates a mechanical painful stimulation by activating group III and group IV muscle nociceptors [3]. Through pressure pain thresholds (PPT), PA provides a quantitative value related to deep structures sensitivity allowing clinicians or researchers to make comparison over time. In case of musculoskeletal pain, as recently proposed in a literature review by Arendt-Nielsen and Yarnitsky [4], PA seems particularly relevant to compare pain over time or between various normal, affected or treated anatomical regions.

It has been reported that pressure pain sensitivity is different between individual muscles [5] and also non uniformly distributed between muscle belly and tendons of a same muscle [6–9]. Thus, according to Anderssen and colleagues [6], the assessment of pain sensitivity in two adjacent sites can lead to two significantly different PPT’s values. This difference could be explained by a change in muscle thickness and density of nociceptors. However, no difference are observed when PPT are assessed bilaterally over homologous body locations [5,10]. Among all the different anatomical locations, the low back region is particularly of interest for PPT’s measurements since 70% of the population will experience Low Back Pain (LBP) at least once in his lifetime [11] and because LBP is often reported in relation to work related musculoskeletal disorders [12], disability and sickness absence from work [13,14]. The assessment of PPT can be used as a method to diagnose and monitor the effectiveness of various treatments or interventions over the lower back region [15–17].

According to a literature review by Arendt-Nielsen and Yarnitsky [4], PA seems relevant to compare pain sensitivity over time or between various normal, affected, or treated anatomical locations. In numerous studies, PA reported good to excellent intra- and inter- reliability to assess pain sensitivity in the low back [16,18–21]. Mokkink and colleagues [22] have defined relative reliability as the extent to which scores for subjects who have not changed are the same for repeated measurements, in our study assessed by one examiner on two different occasions. Relative reliability is commonly quantified using intraclass coefficient correlation (ICC) [23]. Absolute reliability also called “agreement” or “absolute measurement error” is defined as how close the score on repeated measures are [24] and it is quantified using standard error of measurement (SEM). Interestingly, PPT reliability studies have (1) generally assessed only two or four locations over the low back region and/or (2) assessed PPT’s reliability only unilaterally. Further investigations are therefore needed to ensure that PA is a reliable method to assess PPT in numerous locations covering the low back region of asymptomatic individuals.

The purpose of this study study was (1) to evaluate the intra- and inter- absolute and relative reliability of PPT within 14 locations covering the low back region of young asymptomatic individuals and (2) to determine the number of trials required to ensure reliable PPT assessments.

## Materials and Methods

### Subjects

Fifteen asymptomatic subjects (8 women and 7 men), described in Table 1, volunteered to participate in this study. The subjects were recruited within the Grenoble community and consisted of students (11) and newly-hired workers (4). Inclusion criteria were being aged to 18 to 55 years, no musculoskeletal pain in the low back during the last week, no previous injury or/and surgery in the low back region and no pregnancy. This study was conducted in accordance with the Declaration of Helsinki and was approved by the national ethics committee (French society for independent-living technologies and gerontechnology). Subjects gave their informed written consent to the experimental procedure.

**Table 1:** Characteristics of the subjects (n = 15, 8 women and 7 men).

Characteristics	Mean (SD)
**Age (years)**	27.2 (10.2)
**Height (cm)**	170.8 (11.4)
**Weight (kg)**	68.9 (15.6)
**BMI (kg/m²)**	23.3 (2.5)

### Experimental protocol

A Somedic Algometer (Type 2, Sollentuna, Sweden) with a probe size of 1 cm² and calibrated before each session was used to assess PPT over two sessions separated by one hour and lasting approx. 30 minutes. The pressure was applied (1) by a single examiner, (2) over 14 anatomical locations in the lower back region with 7 locations on each side of the lumbar spinal processes L1-L5 and (3) at a rate of 30 kPa/s in line with previous studies. To avoid tissue injury [26–28], a 1 minute interval was observed between two consecutive PPT assessments over the same location to avoid temporal sensitization [29].

Subjects lying comfortably in a prone position were asked to press a button that locks the algometer when the pressure became painful. Then, the examiner noted the pressure indicated on the algometer display corresponding to the PPT. As in numerous studies [30–32] a training PPT measurement was realized prior recordings on the tibialis anterior [33], a remote site from the low back.

### Procedure to mark the 14 anatomical locations

After palpation, the examiner placed two marks at the level of the first (L1) and fifth (L5) vertebrae spinal processes and measures the distance between these two locations (d1). This distance allows the examiner to select one paper grid with 14 anatomical locations among 8 grids specially designed according to the average L1-L5 distance reported earlier [32–33]. Once selected, the examiner aligns the grid with the L1 and L5 marks over the skin and start the experiment.

**Fig 1:**
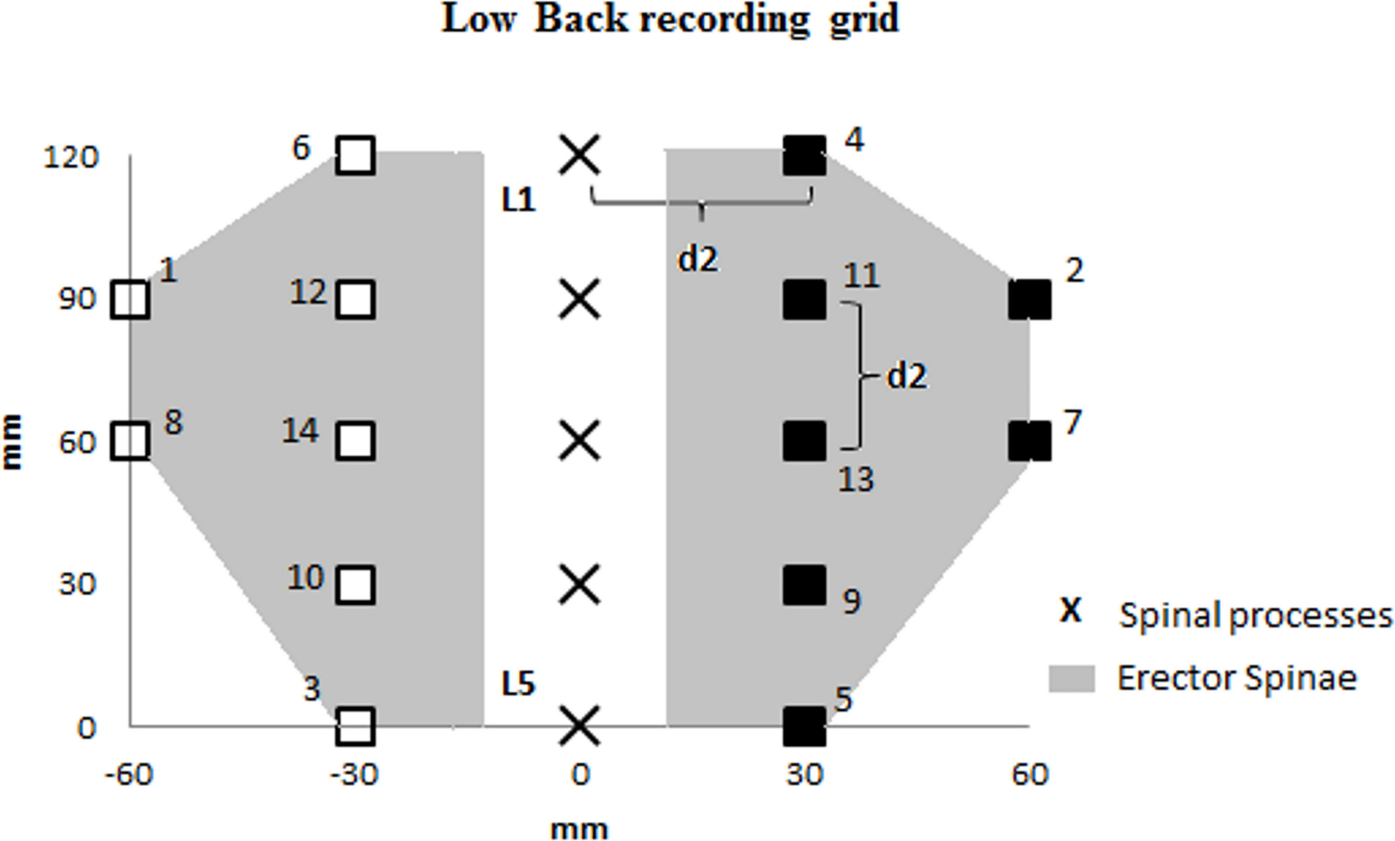
Schematic representation of the low back PPT recording grid of the left (blank square) and right (black squares) erector spinae muscles. The spinal processes of first (L1) to fifth (L5) vertebrae are represented by black crosses.

To design these grids, we calculated d2, corresponding to the quarter of the distance L1-L5. A first column of 5 points was placed bilaterally at the distance (d2) from a fictive line joining L1 to L5. Then, a second column of 2 points was set bilaterally at 2 times the distance (d2) of L2 and L3 (Fig 1).

### Data analysis

PPT measurements were found to be normally distributed (Shapiro-Wilk normality test).

On the one hand, the results of the first session were analyzed using a repeated measure of variance (ANOVA) to investigate the intra-session reliability, followed by Tukey post-hoc test to highlight differences between trials [34]. The relative and absolute reliability across the trials 1-2-3 were computed using ICC, SEM and minimum detectable change (MDC). The relative reliability was evaluated by calculating a 2-way fixed ICC_2,1_ (for absolute agreement). Reliability coefficients (i.e. ICC values) were interpreted according to Landis and Koch [35] in which an ICC between 0.00–0.20 is considered poor, 0.21–0.40 is fair, 0.41–0.60 is moderate, 0.61–0.80 is substantial, and 0.81–1.00 is almost perfect. The SEM expressed in the same unit as pain sensitivity (kPa) quantifies the precision of PPT measurements of individual subjects [23,36]. The SEM was calculated as SD $\sqrt{1-ICC}$ where SD is the standard deviation of the scores from all subjects and ICC the relative reliability [36]. MDC calculated as SEM × 1.96 × $\sqrt{2}$ provides information on the thresholds required to be confident that a difference can be considered as “real” [36]. Mean, standard deviation, ICC_2,1_, SEM, MDC and limits of agreement (LOA) values were calculated for the two sessions to investigate the inter-session reliability in relation to the following three comparisons [16, 37–39]:

1. trial 1 from session 1 *versus* trial 1 from session 2;
2. the mean of trials 1 and 2 (trials 1–2) from session 1 *versus* the mean of trials 1 and 2 (trials 1–2) from session 2;
3. the mean of trials 1,2 and 3 (trials 1-2-3) from session 1 *versus* the mean of trials 1, 2 and 3 (trials 1-2-3) from session 2.

Furthermore, Bland and Altman plots of the differences between trials against their mean and LOA were used to assess the magnitude of disagreement between trials of the 2 sessions. Of note, a difference between trials outside the LOA can be considered as a real change [36].

Finally, one way ANOVA (with the number of trials as within-subject factor) followed by Tukey post-hoc test for pair-wise comparison was performed to compare reliability values (ICC and SEM) of the two sessions.

## Results

### Intra-session reliability

#### Relative reliability of PPT in the low back

As mentioned in Table 2, with values ranged from 0.85 to 0.99, the ICCs of the 14 anatomical locations (Table 2) and of the left, right and overall low-back (P_left_, P_right_, P_all_) were almost perfect regardless of the conducted comparison (trials 1-2-3).

**Table 2:** Intraclass correlation coefficients (ICC), standard error of measurement (SEM) and minimum detectable change (MDC) for pressure pain thresholds assessed during session 1 over 14 locations (P1 to P14) over the low back region, for left and right locations (Pleft and Pright) as well as overall low back (Pall) between the mean of the first and second trials (T1-T2), the first and the third trials (T1-T3), the second and the third trials (T2-T3) and the means of the three trials (T1-T2-T3).

Trials	T1-T2				T1-T3				T2-T3				T1-T2-T3			
	ICC	SEM	MDC	MDC	ICC	SEM	MDC	MDC	ICC	SEM	MDC	MDC	ICC	SEM	MDC	MDC
Points	(95%CI)	(kPa)	(kPa)	(%)	(95%CI)	(kPa)	(kPa)	(%)	(95%CI)	(kPa)	(kPa)	(%)	(95%CI)	(kPa)	(kPa)	(%)
**P1**	0.92 (0.72 - 0.97)	64	179	35	0.88 (0.67 - 0.96)	77	215	42	0.96 (0.88 - 0.99)	44	122	25	0.92 (0.81 - 0.97)	63	175	35
**P2**	0.85 (0.61 - 0.95)	85	234	46	0.90 (0.74 - 0.97)	60	167	33	0.93 (0.81 - 0.98)	59	164	32	0.90 (0.77 - 0.96)	68	190	37
**P3**	0.92 (0.78 - 0.97)	72	200	33	0.91 (0.76 - 0.97)	71	197	31	0.89 (0.71 - 0.96)	85	235	37	0.91 (0.80 - 0.96)	76	210	34
**P4**	0.94 (0.84 - 0.98)	50	138	21	0.86 (0.62 - 0.95)	76	212	33	0.91 (0.74 - 0.97)	67	185	29	0.90 (0.79 - 0.96)	65	180	28
**P5**	0.94 (0.78 - 0.98)	57	159	25	0.91 (0.75 - 0.97)	68	190	30	0.95 (0.86 - 0.98)	52	144	22	0.93 (0.85 - 0.97)	59	164	26
**P6**	0.88 (0.69 - 0.96)	83	229	37	0.88 (0.59 - 0.96)	84	232	37	0.94 (0.83 - 0.98)	60	166	26	0.90 (0.78 - 0.96)	76	210	34
**P7**	0.94 (0.84 - 0.98)	57	159	29	0.90 (0.71 - 0.97)	77	213	38	0.94 (0.83 - 0.98)	60	167	29	0.93 (0.84 - 0.97)	65	180	32
**P8**	0.91 (0.75 - 0.97)	81	224	41	0.91 (0.75 - 0.97)	74	204	37	0.93 (0.81 - 0.98)	65	180	32	0.91 (0.82 - 0.97)	73	202	36
**P9**	0.95 (0.87 - 0.98)	51	142	23	0.86 (0.65 - 0.95)	86	237	37	0.93 (0.81 - 0.98)	64	176	28	0.92 (0.82 - 0.97)	68	188	30
**P10**	0.95 (0.81 - 0.99)	51	141	24	0.96 (0.80 - 0.99)	46	127	22	0.97 (0.91 - 0.99)	40	111	18	0.96 (0.90 - 0.99)	46	126	21
**P11**	0.97 (0.91 - 0.99)	40	112	17	0.94 (0.83 - 0.98)	60	165	26	0.93 (0.81 - 0.98)	61	170	26	0.95 (0.88 - 0.98)	54	150	23
**P12**	0.95 (0.84 - 0.98)	61	168	26	0.98 (0.94 - 0.99)	34	94	15	0.94 (0.81 - 0.98)	62	172	27	0.95 (0.89 - 0.98)	53	148	24
**P13**	0.93 (0.78 - 0.97)	63	174	28	0.88 (0.68 - 0.96)	79	218	35	0.95 (0.87 - 0.98)	45	126	20	0.92 (0.82 - 0.97)	63	176	28
**P14**	0.87 (0.66 - 0.95)	91	253	42	0.89 (0.71 - 0.96)	78	217	37	0.94 (0.84 - 0.98)	57	158	27	0.90 (0.78 - 0.96)	76	212	36
**Pleft**	0.97 (0.91 - 0.99)	41	115	20	0.96 (0.90 - 0.99)	43	118	20	0.99 (0.96 - 1.00)	26	72	12	0.97 (0.94 - 0.99)	37	103	18
**Pright**	0.98 (0.95 - 1.00)	27	75	12	0.95 (0.86 - 0.98)	46	128	21	0.97 (0.93 - 0.99)	35	98	16	0.97 (0.93 - 0.99)	37	102	17
**Pall**	0.98 (0.94 - 0.99)	32	88	15	0.96 (0.88 - 0.99)	44	122	20	0.99 (0.96 - 1.00)	27	74	12	0.97 (0.94 - 0.99)	35	96	16

#### Absolute reliability of PPT in the low back

Table 2 also reports that absolute reliability (.*i*.*e*. SEM) remained non statistically different for all possible combinations. SEM values ranged from 26 to 91 kPa.

#### Number of trials to ensure reliable measurements in the low back

The mean PPT values at each anatomical locations were not significantly different between trials regardless of the three conducted comparisons (trial 1 *versus* trial 2, trial 1 *versus* trial 3, trial 2 *versus* trial 3), the p-values were ranged from 0.7220 to 1.000 (Table 3). Concerning the left, right and overall low-back (P_left_, P_right_, P_all_), p-values ranged from 0.9960 to 0.9995.

**Table 3:** Mean (SD) pressure pain thresholds (kPa) for session 1 assessed over 14 locations covering the low back region and level of significance (p-values) among trial. See “*Procedure to mark the 14 anatomical locations”* for explanation concerning the locations of PPT assessments.

	Trial 1		Trial 2		Trial 3		T1-T2-T3		T1 - T2	T1 - T3	T2 - T3
	Mean	(SD)	Mean	(SD)	Mean	(SD)	Mean	(SD)	p-value	p-value	p-value
**P1**	539.0	(237.3)	487.1	(219.5)	494.4	(209.6)	506.8	(218.2)	0.8114	0.8571	0.9958
**P2**	506.1	(190.2)	512.1	(252.5)	519.2	(205.0)	512.5	(212.2)	0.9971	0.9862	0.9958
**P3**	607.5	(237.7)	621.5	(275.1)	645.7	(244.6)	624.9	(247.2)	0.9883	0.9159	0.9653
**P4**	654.6	(193.9)	651.0	(228.3)	641.3	(214.6)	649.0	(207.6)	0.9989	0.9851	0.9920
**P5**	607.6	(219.8)	653.9	(241.9)	638.9	(233.9)	633.4	(227.2)	0.8583	0.9324	0.9840
**P6**	593.4	(231.9)	626.6	(249.0)	664.6	(249.7)	628.2	(239.5)	0.9312	0.7220	0.9107
**P7**	534.9	(243.0)	565.8	(250.7)	586.0	(246.9)	562.2	(241.7)	0.9413	0.8481	0.9745
**P8**	535.5	(259.3)	564.0	(278.9)	571.6	(227.3)	557.0	(250.2)	0.9534	0.9264	0.9966
**P9**	629.8	(227.8)	620.0	(259.1)	657.6	(241.3)	635.8	(237.6)	0.9938	0.9506	0.9118
**P10**	560.9	(246.8)	605.6	(238.4)	604.6	(230.5)	590.4	(233.7)	0.8734	0.8790	0.9999
**P11**	628.9	(242.4)	651.4	(216.1)	664.1	(251.9)	648.1	(231.9)	0.9659	0.9187	0.9890
**P12**	616.0	(263.2)	654.4	(262.7)	616.5	(229.4)	629.0	(246.7)	0.9145	1.0000	0.9166
**P13**	596.9	(243.6)	638.6	(222.5)	634.9	(212.1)	623.5	(221.7)	0.8773	0.8969	0.9990
**P14**	604.2	(252.5)	593.3	(264.2)	573.4	(221.4)	590.3	(240.9)	0.9925	0.9418	0.9753
**Pleft**	579.5	(235.0)	593.2	(240.4)	595.8	(218.2)	589.5	(225.8)	0.9865	0.9810	0.9995
**Pright**	594.1	(211.0)	613.2	(229.5)	620.3	(215.8)	609.2	(213.8)	0.9709	0.9463	0.9960
**Pall**	586.8	(220.8)	603.2	(233.6)	608.1	(214.5)	599.4	(217.8)	0.9793	0.9656	0.9982

The comparison of means of ICC regardless of the conducted comparisons between trials (Table 2) showed a statistical difference for Trials 2–3 *versus* Trials 1–3 (p = 0.0338). The same analysis for SEM further showed no significant difference between trials.

### Inter-session reliability

#### Relative reliability of PPT in the low back

**Fig 2:**
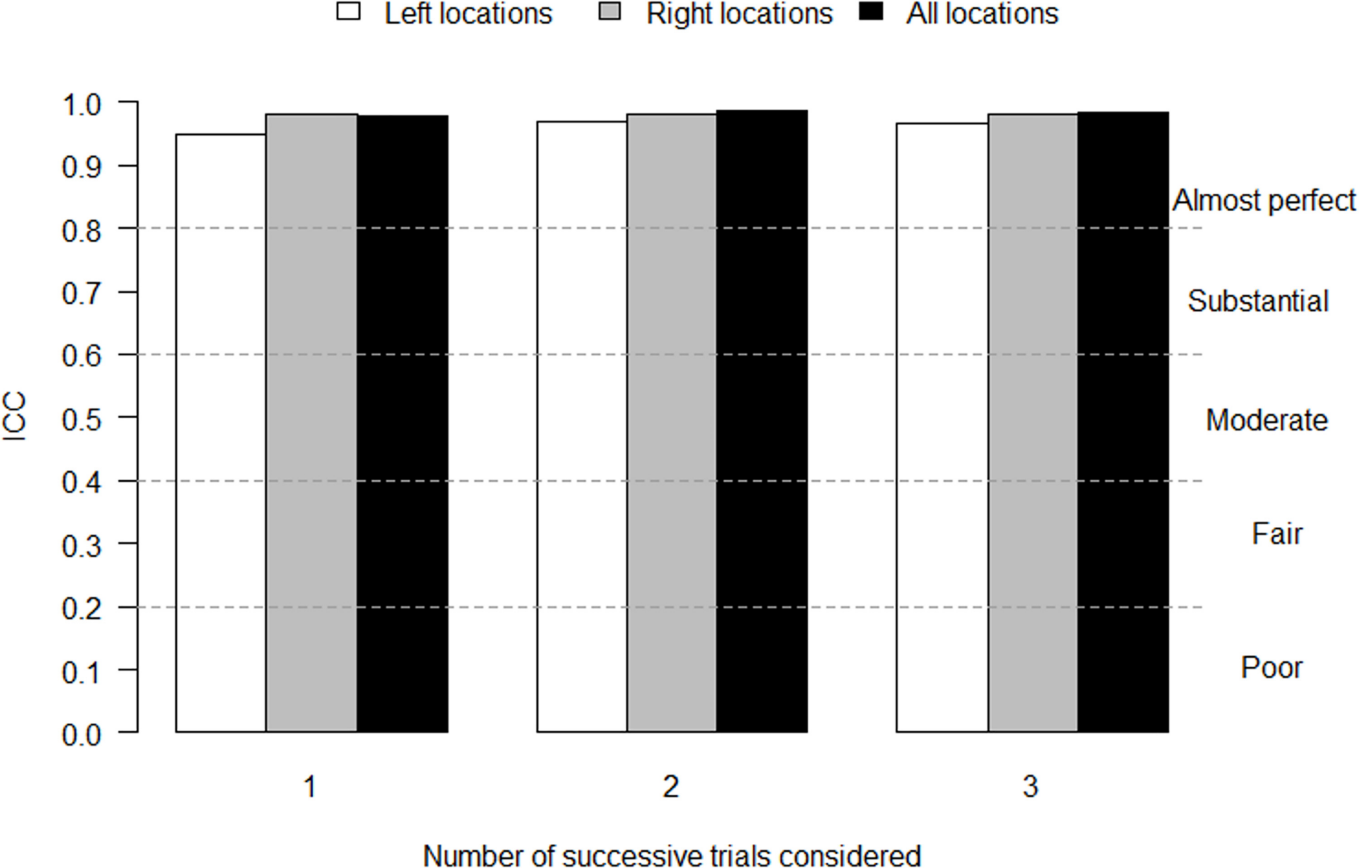
Inter-session ICC values of the left, right and all locations depending on the number of successive trials considered.

The ICCs of the 14 locations were almost perfect regardless of the conducted comparison (Table 4 and Fig 2). ICC values ranged from 0.86 to 0.99.

**Table 4:** Mean (±SD); Standard Error of Measurement (SEM); Minimal Detectable Change (MDC); Intraclass Correlation Coefficient (ICC), 95% Confidence Interval (CI) of ICC, and 95% Limits of Agreement (LoA) for Pressure Pain Threshold (PPT) among 14 lumbar locations, left locations (P_left_), right locations (P_right_) point and all the locations (P_all_) when 1,2 or 3 trials during session 1 and session 2 are compared.

Number of trials	Points	Mean	Mean	SEM	MDC	MDC	ICC	95% LOA	p-value
		Session 1 (SD)	Session 2 (SD)	(kPa)	(kPa)	(%)	(95% CI)		
**1**	**Point 1**	553.5 (235.4)	530.2 (167.7)	75	209	39	0.86 (0.64 - 0.95)	-190.89 - 237.42	0.7575
	**Point 2**	520.7 (191.8)	518.4 (194.7)	67	186	36	0.87 (0.67 - 0.96)	-192.77 - 197.43	0.9739
	**Point 3**	633.7 (250.5)	660.7 (251.3)	77	212	33	0.90 (0.74 - 0.97)	-243.45 - 189.32	0.7698
	**Point 4**	677.6 (207.1)	678.4 (233.3)	43	119	18	0.96 (0.89 - 0.99)	-125.62 - 124.02	0.9921
	**Point 5**	633.7 (234.8)	648.0 (254.9)	74	206	32	0.90 (0.74 - 0.97)	-228.74 - 200.21	0.2770
	**Point 6**	620.5 (246.9)	634.5 (249.9)	89	247	39	0.87 (0.65 - 0.95)	-271.13 - 243.13	0.8784
	**Point 7**	534.9 (234.1)	536.7 (221.2)	60	165	31	0.93 (0.80 - 0.98)	-175.36 - 171.63	0.9822
	**Point 8**	547.4 (254.1)	562.2 (222.2)	74	206	37	0.90 (0.73 - 0.97)	-228.71 - 199.11	0.8664
	**Point 9**	654.5 (239.4)	645.5 (234.1)	80	222	34	0.88 (0.68 - 0.96)	-223.79 - 241.66	0.9184
	**Point 10**	590.1 (263.5)	637.6 (240.0)	54	150	24	0.95 (0.79 - 0.99)	-173.19 - 78.25	0.6100
	**Point 11**	653.6 (252.5)	669.1 (232.5)	65	179	27	0.93 (0.80 - 0.97)	-201.48 - 170.42	0.8621
	**Point 12**	641.6 (272.3)	673.0 (258.6)	52	145	22	0.96 (0.88 - 0.99)	-169.82 - 107.02	0.7485
	**Point 13**	623.7 (256.8)	661.5 (237.2)	95	262	41	0.85 (0.62 - 0.95)	-302.81 - 227.21	0.6785
	**Point 14**	630.6 (263.9)	643.9 (238.9)	89	248	39	0.87 (0.66 - 0.95)	-271.63 - 244.97	0.8857
	**Pleft**	602.5 (243.3)	620.3 (219.3)	51	143	23	0.95 (0.86 - 0.98)	-163.59 - 127.94	0.8346
	**Pright**	614.1 (217.6)	622.5 (216.4)	37	102	17	0.91 (0.76 - 0.97)	-201.86 - 142.34	0.7008
	**Pall**	608.3 (228.5)	621.4 (216.8)	41	114	19	0.95 (0.85 - 0.98)	-159.01 - 111.42	0.7660
**2**	**Point 1**	533.5 (230.7)	532.2 (178.0)	53	147	28	0.93 (0.81 - .098)	-152.94 - 155.48	0.9867
	**Point 2**	532.7 (226.0)	536.1 (203.2)	55	153	29	0.93 (0.81 - 0.98)	-163.63 - 156.76	0.9654
	**Point 3**	637.8 (258.6)	646.9 (259.2)	52	146	23	0.96 (0.88 - 0.99)	-161.09 - 142.83	0.9237
	**Point 4**	675.9 (220.0)	700.1 (234.5)	47	130	19	0.96 (0.88 - 0.98)	-151.96 - 103.63	0.7731
	**Point 5**	655.3 (239.8)	659.4 (233.0)	63	175	27	0.93 (0.79 - 0.97)	-188.14 - 180.01	0.4137
	**Point 6**	636.0 (246.3)	657.3 (261.4)	64	177	27	0.93 (0.82 - 0.98)	-202.58 - 159.91	0.8197
	**Point 7**	552.7 (234.9)	549.1 (224.6)	52	143	26	0.95 (0.85 - 0.98)	-147.01 - 154.34	0.9655
	**Point 8**	562.1 (257.8)	566.0 (227.9)	45	124	22	0.97 (0.90 - 0.99)	-133.75 - 125.89	0.9650
	**Point 9**	649.9 (251.6)	661.2 (237.5)	50	137	21	0.96 (0.88 - 0.99)	-153.98 - 131.38	0.9002
	**Point 10**	611.0 (255.7)	621.7 (234.4)	32	89	15	0.98 (0.95 - 0.99)	-102.29 - 80.89	0.9057
	**Point 11**	664.1 (238.5)	670.7 (233.9)	65	179	27	0.92 (0.79 - 0.97)	-194.01 - 180.88	0.9399
	**Point 12**	659.5 (267.4)	665.6 (249.8)	41	112	17	0.97 (0.93 - 0.99)	-123.84 - 111.64	0.9490
	**Point 13**	643.2 (242.2)	673.0 (234.4)	62	171	26	0.93 (0.81 - 0.98)	-198.91 - 139.31	0.7345
	**Point 14**	625.5 (261.7)	641.9 (236.6)	57	158	25	0.95 (0.85 - 0.98)	-179.27 - 146.53	0.8587
	**Pleft**	609.3 (244.0)	618.8 (225.3)	33	92	15	0.98 (0.94 - 0.99)	-103.89 - 84.94	0.9128
	**Pright**	624.8 (227.1)	635.7 (218.2)	31	87	14	0.98 (0.94 - 0.99)	-99.62 - 78	0.7887
	**Pall**	617.1 (234.2)	627.2 (220.6)	30	84	14	0.98 (0.95 - 0.99)	-96.20 - 75.92	0.8514
**3**	**Point 1**	526.7 (222.6)	537.8 (177.0)	49	136	26	0.94 (0.83 - 0.98)	-152.32 -130.14	0.8810
	**Point 2**	537.3 (223.6)	531.4 (193.2)	48	134	25	0.94 (0.84 - 0.98)	-134.61 -146.47	0.9386
	**Point 3**	648.3 (252.7)	655.7 (255.8)	48	134	21	0.96 (0.89 - 0.99)	-147.88 -133.03	0.9368
	**Point 4**	672.4 (217.6)	701.2 (235.8)	43	118	17	0.96 (0.88 - 0.99)	-138.51 -80.73	0.7300
	**Point 5**	657.9 (238.5)	662.4 (234.2)	54	150	23	0.95 (0.85 - 0.98)	-161.65 - 152.58	0.5269
	**Point 6**	653.0 (247.2)	661.0 (257.8)	57	157	24	0.95 (0.85 - 0.98)	-172.42 - 156.28	0.9309
	**Point 7**	563.6 (232.6)	544.8 (213.2)	50	139	25	0.95 (0.86 - 0.98)	-122.04 - 159.60	0.8194
	**Point 8**	569.8 (244.6)	574.7 (227.3)	42	116	20	0.97 (0.91 - 0.99)	-126.58 - 116.63	0.9544
	**Point 9**	660.1 (246.4)	667.0 (230.8)	39	107	16	0.97 (0.92 - 0.99)	-118.44 - 104.66	0.9376
	**Point 10**	617.7 (251.1)	632.5 (232.9)	37	104	17	0.98 (0.93 - 0.99)	-119.91 - 90.17	0.8677
	**Point 11**	671.6 (242.2)	671.3 (239.3)	54	149	22	0.95 (0.85 - 0.98)	-156.27 - 156.85	0.9974
	**Point 12**	653.7 (258.0)	672.2 (247.5)	45	124	19	0.97 (0.91 - 0.99)	-144.05 - 107.16	0.8431
	**Point 13**	648.6 (233.7)	674.1 (229.4)	42	116	18	0.97 (0.90 - 0.99)	-136.42 - 85.35	0.7649
	**Point 14**	617.6 (252.6)	647.8 (237.9)	52	145	23	0.95 (0.86 - 0.98)	-170.55 - 110.15	0.7386
	**Pleft**	612.4 (238.0)	626.0 (224.2)	34	94	15	0.98 (0.94 - 0.99)	-108.19 - 81.03	0.8734
	**Pright**	630.2 (224.2)	636.0 (215.8)	26	71	11	0.99 (0.96 - 1.00)	-79.86 - 68.19	0.8708
	**Pall**	621.3 (229.5)	631.0 (219.0)	28	77	12	0.98 (0.95 - 0.99)	-88.38 - 68.96	0.8711

**Fig 3:**
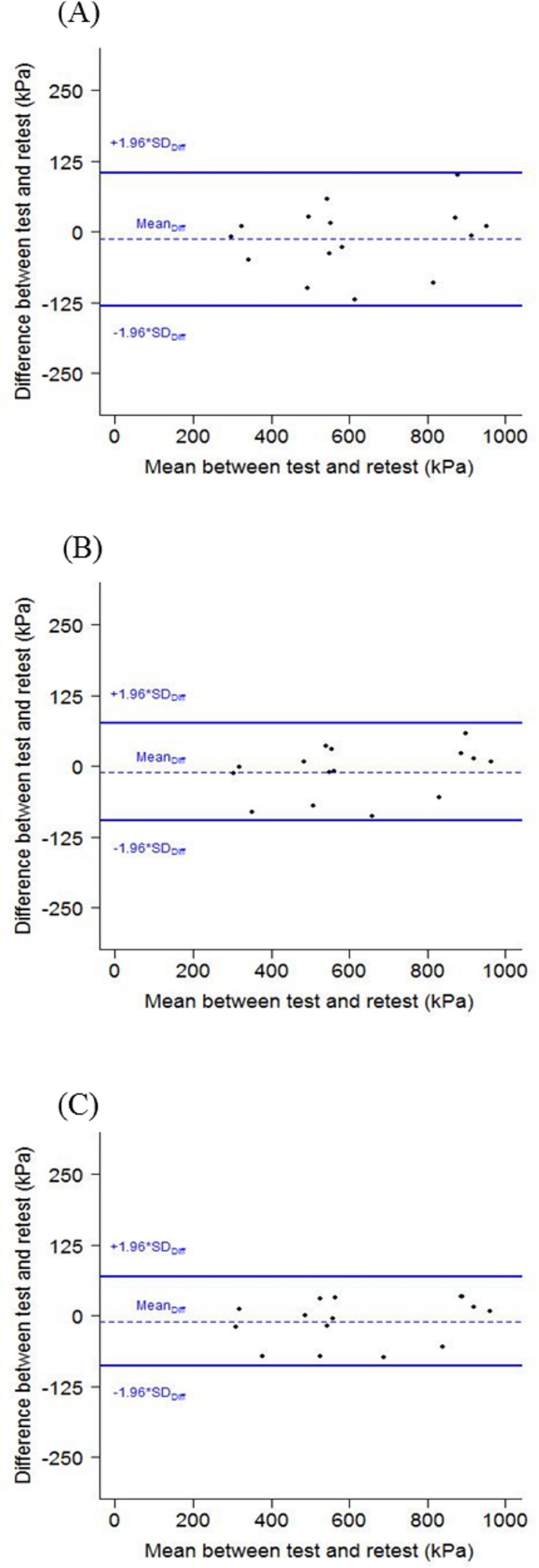
Bland and Altman analyses plotted for worker’s pressure pain threshold of the overall low back for the mean of the first trials (A), the first and second trials (B) and the three trials(C).

The visual analysis of Bland and Altman’s plots suggested no difference in PPT values between sessions because (1) zero was included in the 95% confidence interval and (2) all the subjects were inside the limits of agreement (Fig 3). Furthermore, this visual analysis also suggested narrowed LOA for the association trials 1–2 and trials 1-2-3 compared to the plot of the first trial.

#### Number of trials to ensure reliable measurements in the low back

The mean PPT values at each pressure pain location between sessions 1 and 2 were not significantly different regardless of the following three comparisons: (1) trial 1 from session 1 *versus* trial 1 from session 2, (2) the mean of trials 1 and 2 (trials 1–2) from session 1 *versus* the mean of trials 1 and 2 (trials 1–2) from session 2, (3) the mean of trials 1, 2 and 3 (trials 1-2-3) from session 1 *versus* the mean of trials 1, 2 and 3 (trials 1-2-3) from session 2, the p-values were ranged from 0.4137 to 0.9974.

**Fig 4:**
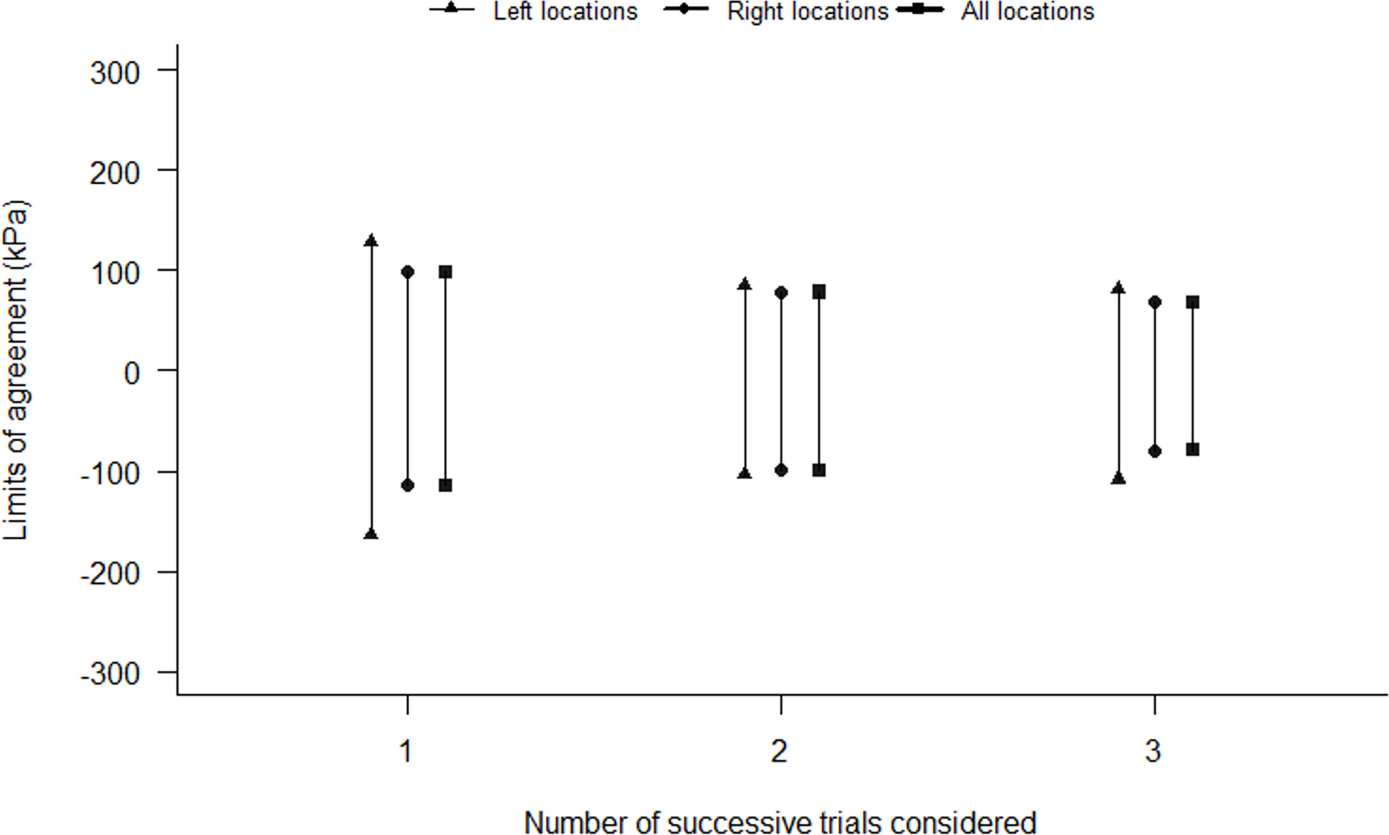
Limits of Agreement (LoA) values of left, right and the overall back locations are averaged to calculate PPTs measurements.

When two consecutive measurements (Trials 1–2 or Trials 2–3) or all trials were used to calculate subjects’ relative and absolute reliability, ICC and SEM values were significantly higher than when the first trial was used. Conversely, no statistical difference was observed between the two first consecutive trials and all trials, or between the two last consecutive trials and all trials. Finally, no statistical difference was observed between the two first and the two last consecutive measurements (Tables 5 and 6). Visual analysis of Bland and Altman plots showed that LOA values decreased when the two first and the three trials were analyzed (Fig 4).

**Table 5:** Comparison of means of ICCs values regardless of the conducted comparisons between trials.

Comparaison	Difference (95% CI)	p-value
**Trials 1–2 *vs*. Trial 1**	0.040 (0.015 - 0.064)	0.0004***
**Trials 2–3 *vs*. Trial 1**	0.043 (0.018 - 0.068)	0.0002***
**Trials 1-2-3 *vs*. Trials 1**	0.043 (0.019 - 0.068)	0.0001***
**Trials 2–3 *vs*. Trials 1–2**	0.025 (0.023 - 0.028)	0.9937
**Trials 1-2-3 *vs*. Trials 1–2**	0.003 (0.021 - 0.028)	0.9431
**Trials 1-2-3 *vs*. Trials 2–3**	0.001 (0.024 - 0.026)	0.9998

*p-value<0.05

** p-value<0.01;

*** p-value<0.001.

Abbreviations: ICC, Intraclass Correlation Coefficient; CI, Confidence Interval.

**Table 6:** Comparison of means of SEMs values regardless of the conducted comparisons between trials.

Comparaison	Difference (95% CI)	p-value
**Trials 1–2 *vs*. Trials 1**	-17.222 (-29.523 - -4.920)	0.0025**
**Trials 2–3 *vs*. Trial 1**	-18.835 (-31.136 - -6.634)	0.0008***
**Trials 1-2-3 *vs*. Trials 1**	-19.081 (-31.383 - -6.780)	0.0007***
**Trials 2–3 *vs*. Trials 1–2**	-1.613 (-13.915 - 10.688)	0.9856
**Trials 1-2-3 *vs*. Trials 1–2**	-1.859 (-13.460 - 9.741)	0.9207
**Trials 1-2-3 *vs*. Trials 2–3**	0.246 (-12.548 - 12.056)	0.9999

*p-value<0.05

** p-value<0.01;

*** p-value<0.001.

Abbreviations: ICC, Intraclass Correlation Coefficient; CI, Confidence Interval.

## Discussion

Considering the importance of collecting reliable PPT over the lower back region, the purpose of the present experiment was (1) to evaluate the intra- and inter- absolute and relative reliability of PPT within 14 locations covering the low back region of asymptomatic individuals, and (2) to determine the number of trial required to ensure reliable PPT assessments. Intra-session results will be discussed before those of the inter-session.

First, the analysis of PPT measurements of the low back showed excellent relative reliability for the intra-session. ICCs values were almost perfect regardless of the conducted comparisons (trial 1 *versus* trial 2, trial 1 *versus* trial 3, trial 2 *versus* trial 3) suggesting no difference in PPTs’ measurements between trials and no systematic error in the data. Moderate to excellent relative reliability was also obtained in previous studies, assessing PPT in other anatomical locations such as tibia [40], calf, hand [41] and trapezius [42]. In a recent study assessing PPT in the lower back region of young healthy subjects, Waller and colleagues [43] reported ICC ranged from 0.94 to 0.99 and further conclude that intra-rater reliability was excellent in the low back. As Waller and colleagues [43] have assessed PPT only over one location in the low back (2 cm laterally from L4/L5). However, the generalization of such a finding is questionable considering that PPT can be different over the same muscle [6]. Moreover, the study population was small but sufficient to obtain substantial relative reliability values [35].

The inter-session relative reliability has also been shown to be excellent in our study. It is first important to note that no significant difference was observed for PPT measurements between session 1 and session 2. Then, the analysis trial-to-trial showed that ICCs values were also almost perfect regardless of the number of trials considered, confirming excellent reliability previously reported by Koo and colleagues [19] in the low back of healthy individuals. In the latter study, the six anatomical locations assessed (1) bilaterally in the low back perpendicularly to the spinal processes of L1, L3 and L5 and (2) over two sessions separated by 5 minutes led to ICC ranged from 0.86 to 0.91. Conversely to the intra-session’s results, we report higher relative and absolute inter-session reliability (*i*.*e*. ICC and SEM values) when two consecutive PPTs measurements were used for data analysis. Similar results have also been reported in the low back of healthy individuals by Chesterton and colleagues [29], i.e, higher intra-session reliability for the mean of three consecutive PPTs assessments than when only the first assessment was used for analysis. Even more interesting to note was that in our study, contrary to numerous studies [18,31,44], the first PPT assessment did not need to be discarded to obtain excellent reliability for both intra- and inter- session. Lacourt and colleagues [44], in a test-retest study have reported significant differences in PPTs values respectively, between the first and second PPT measurement and also between the first and third one. This result led them to use only the second and third PPT measurements for data analysis. Higher inter-session reliability was also found by Nussbaum and Downes [31], when the first PPT measurement was omitted. This could be explained by the effort made in the current study to familiarize the subject with PPT measurements (tests at a remote location, one practice session).

As ICC is largely influenced by between-subjects variability and does not provide information on typical error [36], it was necessary to complete our analysis by computing SEM and MDC. When the first PPT measurement was associated with the second or third one, SEM were generally below 65 kPa and MDC ranged from 11% to 27% (71 kPa to 179 kPa). In other words, this result suggests that (1) the true score of PPT was 65 kPa below or above the observed score and (2) that a clinical change will not be masked by measurement error if the observed score changed by more than 11 to 27%. The limited number of published studies assessing absolute reliability has made comparison difficult with the existing literature. However, after two sessions of PPTs measurements over one location on the trapezius muscle and the tibialis anterior separated by three to five days, Walton and colleagues [45] have reported a SEM value close to ours with a value of 49 kPa. Similar results were found by Fingleton and colleagues [25] in the lower limbs with SEM ranged from 16 to 39 kPa and by Chesterton and colleagues [29] in the back with SEM equal to 60 kPa.

In general, when looking at the number of trials required ensuring reliable measurement in the low back, our results are rather original. Indeed, we have reported almost perfect intra- and inter-session reliability on the first PPT measurement (ICC ranged from 0.85 to 0.99) suggesting that one training trial over the tibialis anterior would be sufficient to familiarize the participant with the PPT procedure. Hopkins in 2000 [46] assumed that the reliability of a test could be influenced by several factors such as motivation or boredom. For instance, during series of trials, the second one is often better than the first because participants want to improve their performance or because they benefit from the experience of the first one. Conversely, a decreased performance between the first trial and the following ones could be explained by fatigue or loss of motivation. In our study it seems that there is no learning effect between trials because PPT values did not change. Then, it seems that the cognitive and attentional resources needed to perform three consecutive PPT assessments over the low back do not generate boredom or loss of motivation and do not influence reliability. Furthermore, even though both relative and absolute relative reliabilities were significantly higher when two or three consecutive measurements were used for data analysis compared with the first measurement, no statistical difference was observed between the two first and two last PPT measurements. Therefore, this result suggests that using the two first PPT measurements for data analysis will not lead to lower relative and absolute reliabilities than using the two last or three PPT measurements. Finally, in accordance with existing literature in the low back [16,45], MDC values were regularly between 100 and 200 kPa corresponding to approx. 10–20% of the PPT scale range considered as acceptable measurement error by Chiarotto and colleagues [47].

For instance, to be confident that a true change was observed in the low back of young football player after an intervention, Madeleine and colleagues [16] reported MDC value of 140 kPa. Walton and colleagues [45] assessing PPTs in the trapezius muscle among young healthy subjects reported MDC of 113 kPa. These results imply a small sensitivity to change and that a change in PPT measurement can be masked by the measurement error regardless of the absolute changes in PPT [16].

Recent studies have reported good to excellent PPTs’ reliability between sessions, respectively separated by one day and assessed over 14 locations covering the abdominal region [48], two days and assessed over the 2 locations from the low back [20] and twenty-one days and assessed 1 location from the trapezius muscle [49]. Still, the current results need to be confirmed by assessing PPT’s reliability over longer period of time. Then, the absence of significant difference for some important parameters such as ICC and SEM values between two or three consecutive PPT measurements could be explained by the relatively small sample size. Indeed, true significant effects might have been missed in our study because the sample size used might have not adequate power for detecting a true difference of a meaningful magnitude.

Finally, we recruited a mixed population of asymptomatic individuals classified as such since they did not report pain in the low-back within the last 7 days prior to the experiment and had no history of low back injury or/and surgery. However, pain is usually fluctuating as reported by recent studies [50,51,52]. Further, the present results should not be generalized to specific population or gender as gender differences are reported in pressure pain sensitivity [33,53,54]. Still, Paungmali and colleagues [20] have reported almost perfect relative reliability for chronic non-specific low back pain individuals in line with our results. Future studies could address the relative and absolute variability of PPT assessed over 14 locations covering the low back in population suffering from LBP.

## Conclusions

Excellent relative and absolute reliability of PPT measured over 14 locations covering the low back of asymptomatic individuals were reported for both intra- and inter-session. Reliable measurements can be equally achieved when using the mean of consecutive PPT measurement or with only the first one. Although reliability was almost perfect regardless of the conducted comparison between PPT assessments, our results suggest using at least two consecutive measurements to obtain higher inter-session absolute reliability among asymptomatic participants in the low back region. Further studies are needed to enable a more global generalization of these findings.
